# Cloud accounting adoption in Thai SMEs amid the COVID-19 pandemic: an explanatory case study

**DOI:** 10.1186/s13731-022-00234-3

**Published:** 2022-06-21

**Authors:** Dalinee Sastararuji, Danupol Hoonsopon, Pongsakorn Pitchayadol, Pimsiri Chiwamit

**Affiliations:** 1 Technopreneurship and Innovation Management Program, Graduate School, Chulalongkorn University, Bangkok, Thailand; 2 Chulalongkorn Business School, Chulalongkorn University, Bangkok, Thailand; 3 Faculty of Commerce and Management, Prince of Songkla University, Hat Yai, Thailand

**Keywords:** Cloud accounting, Technology adoption, SMEs, Technology–organization–environment framework, Diffusion of innovation theory, Institutional theory

## Abstract

The COVID-19 pandemic has altered the way small and medium-sized businesses (SMEs) function. To maintain business efficiency and reduce operating costs in the context of the constraints imposed by the pandemic, SMEs have been forced to embrace new digital technologies. Cloud accounting is becoming an increasingly important business operating tool for SMEs. By adopting cloud-based accounting, SMEs can become more efficient, financially organized, and flexible. This study aims to investigate the factors that have a pervasive influence on cloud accounting adoption among SMEs in Thailand, following the pandemic's effects. This study integrates three technology adoption theories—the Technology–Organization–Environment framework, Diffusion of Innovation theory, and Institutional Theory—and studies them alongside SMEs' unique characteristics. The research employs a qualitative case study method and triangulated sources of evidence. The findings provide important implications for the research community, policymakers, cloud accounting vendors, and SME owners aiming to formulate better approaches to cloud accounting adoption after the pandemic. The results suggest that vendors should focus on SMEs' particular characteristics and needs. By contrast, SMEs should determine the organizational fit of the cloud accounting platform and integrate cloud accounting with other aspects of their operations.

## Introduction

Small- and medium-sized enterprises (SMEs) are commonly considered able to drive a country's economic growth, competitiveness, and innovation (Halabí & Lussier, 2014; Pitchayadol et al., 2018). In most countries, SMEs account for approximately 90% of all private sector businesses, over 50% of private sector employment, and up to 40% of private sector turnover (World Bank, 2019). In early 2020, the COVID-19 outbreak caused major disruption to all aspects of society and it is likely to have a long-term impact on SMEs' operation. Some SMEs, especially in the tourism, trade, education, transportation, and health sectors, have been dealing with faltering revenue and a changing business landscape (Albulescu, 2020). In Thailand, the second country after China to be affected by COVID-19, lockdown enforcement and physical distancing has had an unprecedented and severe impact on all aspects of how SMEs work, produce, trade, and communicate.

Under these restrictions, SMEs are more vulnerable than large and global enterprises (Kumar & Ayedee, 2021; Papadopoulos et al., 2020). One reason for this is that they typically lag in the adoption of digital technologies, which can help build resilience in the current and post-COVID-19 business environment (Fitriasari, 2020; Organization for Economic Cooperation & Development, 2021). In Japan, for instance, there was a gap in the prevalence of teleworking between larger corporations (48%) and SMEs (10–20%) even as companies sought to deal with the crisis. Nevertheless, recent surveys show that the pandemic has dramatically increased the use of digital technologies by SMEs to ensure business continuity and societal resilience, although considerable differences exist across countries. For instance, 70% of SMEs in the Asia Pacific are accelerating their digitalization, such as through cloud computing, in response to COVID-19, whereas lower-middle-income countries in other regions show 10 times lower technology usage during COVID-19 due to lower Internet penetration rates and high affordability barriers (Idc-Cisco, 2020; Strusani & Houngbonon, 2020). According to UNIDO (2020), a recent survey indicated that among all firms in Thailand, SMEs—especially low-tech firms—have suffered most from extreme revenue loss and movement restrictions, and have least access to government support.

Since finance forms the backbone of SMEs, there is an increased need across verticals for a new type of accounting—one that is more timely, automated, and provides remote access and intelligent analysis. In the post-COVID-19 world, cloud accounting can help SMEs by providing an online platform that includes real-time financial, inventory, workflow, and sales and expense data at affordable rates (Winarsih et al., 2021). However, awareness regarding and usage of cloud accounting in Thailand is still limited. Moreover, there is a gap in the current knowledge, particularly from evidence-based studies, on the use of cloud-based technology in response to disruptive events, such as COVID-19 (Papadopoulos et al., 2020).

Therefore, in this paper, we explore cloud accounting adoption in response to COVID-19's destructive challenges, using SMEs in Thailand as a case study. The key research questions that this study aims to answer are: [i] Why do SMEs adopt cloud accounting? [ii] How do SMEs start adopting cloud accounting? and [iii] What are the factors driving SMEs’ adoption of cloud accounting? By integrating relevant theoretical and conceptual underpinnings, this explanatory case study deepens academic and practical insight into the cloud accounting adoption process in SMEs, in response to the impact of COVID-19.

## Literature review

### SMEs and COVID-19

The definition of SMEs still varies across countries and industries, and is usually based on quantitative criteria, such as the number of employees, capital, assets, and annual revenue (Harvie & Lee, 2002; Pitchayadol et al., 2018). For instance, Jordanian law defines SMEs as firms with less than 100 full-time employees (Dmour et al., 2016). By contrast, Thai SMEs were recently redefined as enterprises with no more than 200 employees and annual revenue of up to 500 million THB based on their sector, as shown in Table 1.

**Table 1:** Classification of enterprise size in Thailand

Type	Manufacturing		Trading and service	
	No. of employees	Annual revenue (THB)	No. of employees	Annual revenue (THB)
Micro	1–5	< 1.8 mil	1–5	< 1.8 mil
Small	6–50	> 1.8 mil–100 mil	6–30	> 1.8 mil–5 mil
Medium	50–200	> 100 mil–500 mil	31–100	> 50 mil–300 mil

Source: The Office of SMEs Promotion (2020)

Although no universally accepted definition of SMEs exists, some common characteristics include small size, limited resources, a simple business structure, and owner dependence (Berisha & Pula, 2015; Lopez-Nicolas & Soto-Acosta, 2010). Most SMEs are owned by a single person and thus, most decision-making—including that concerning investment and liability—is vested in the sole owner as their lifelong duty (Loecher, 2000; Oni & Papazafeiropoulou, 2014).

Before the COVID-19 pandemic, the information technology (IT) adoption rate of SMEs was traditionally low, and the failure rate high (Mole et al., 2004; Nguyen et al., 2015). Multiple studies reveal that SMEs' IT adoption is inhibited by internal barriers, such as the owner‐manager's characteristics, users' resistance to change, limited technological resources, and business size, as well as external factors, such as legal, regulatory, social, cultural, and economic barriers (Chouki et al., 2020; Zaied, 2012). In addition, lack of IT planning and absence of IT knowledge are among the leading causes of SMEs' failure (Bull, 2003; Levy et al., 2001; Puriwat & Hoonsopon, 2021).

Due to the COVID-19 crisis, SMEs are facing new challenges, including severe revenue loss, a sharp decrease in labor supply, mandatory telework, and rapid movement to digital sales and e-payment (International Trade Center, 2020). The COVID-19 outbreak has made the SME owner‐manager more aware of the need for IT adoption. The shift in SMEs' IT adoption priorities, as observed in the literature, are summarized in Table 2.

**Table 2:** Evidence in support of SMEs' IT adoption during COVID-19

Authors	Technology topic	Evidence
Kumar and Ayedee (2021)	Technology 4.0, e.g., social media, e-commerce, and cloud	The adoption of technology 4.0. can help identify customer demands, increase sales and turnover, maintain physical distance, and increase automation
Fitriasari (2020)	Digital transformation	SMEs' business resilience is aided by digital transformation, which occurs when new digital skills develop, and digital tools are embraced
Papadopoulos et al. (2020)	Digital technologies, e.g., mobile, collaborative technologies, big data analytics, IoT, AI, and blockchain	In response to COVID-19, digital technology should be exploited for securing business continuity for SMEs during intense disruptions and global community shocks
Strusani and Houngbonon (2020)	Cloud, big data analytics, AI, robotics, IoT, blockchain, drones, 3D printing, genomics, and distributed power systems, favored by 5G	Digitalization of business processes, with increased use of automation, digital financial services, remote-control systems, and additive manufacturing are critical in the COVID-19 era

### Cloud accounting adoption

Accounting plays a vital role in the success or failure of businesses of all sizes; thus, accounting software was developed to help businesses stay financially organized (Marand et al., 2013). Traditionally, accounting software is purchased as a product and installed locally on an individual user's desktop computer (Dimitriu & Matei, 2015). Cloud accounting, in contrast, provides on-demand accounting services with anytime access to multiple users from anywhere, through the vendor's Internet-based applications (Christauskas & Miseviciene, 2012). In recent years, cloud computing, together with artificial intelligence (AI), big data analytics, and blockchain, have dramatically transformed the entire accounting process and business environment (Ionescu, 2019; Wattana Viriyasitavat & Hoonsopon, 2019; Viriyasitavat et al., 2019; Yoon, 2020).

Cloud accounting is also known as “online accounting,” “web accounting,” “e-accounting,” “virtual accounting system,” “real-time accounting,” or “cloud accounting software” (Christauskas & Miseviciene, 2012; Đorđević et al., 2018; Ionescu, 2019). Cloud accounting differs from traditional accounting in multiple areas, such as the software license type (rent vs buy), system location (cloud vs user's site), and maintenance and support fees (included in package vs requiring extra purchase). Cloud accounting has emerged with the convergence of the basic principles of cloud computing and the activities of the accounting information system (Khanom, 2017). Its key functions consist of collecting and storing financial activities, transforming data into valuable information for decision makers, and controlling the reliability of information (Christauskas & Miseviciene, 2012; Romney et al., 2012).

However, few studies have investigated cloud accounting adoption from the SME business perspective. Most research on cloud computing has been experimental in nature, focused more on technical issues, and have not applied relevant theory (Senyo et al., 2018). Moreover, few studies have been undertaken in emerging markets (Kim et al., 2017). In addition, the COVID-19 pandemic has permanently changed consumer behaviors and the business landscape, and therefore, it is important to investigate the attitude of SMEs toward cloud accounting adoption, as it could play a critical role in the success or failure of SMEs.

### Technology‐organization‐environment (TOE) framework

The technology–organization–environment (TOE) framework, formulated by Tornatzky et al. (1990) identifies three facets that affect a firm's information technology/information systems (IT/IS) adoption: technological, organizational, and environmental. The technological context is the pool of internal and external technologies that are useful to the firm. Rogers' (2010) five perceived characteristics of innovation, namely, relative advantage, complexity, observability, compatibility, and trialability are considered part of this context (Van de Weerd et al., 2016). The organizational context refers to key resources and the nature of the business, such as scope, size, firm structure, and employee culture (Rahayu & Day, 2015). The environmental context involves pressures and supports that exist in the business field, such as customers, competitors, the market, business partners, regulators, and infrastructure. In the SME context, TOE offers a holistic framework supported by theory-consistent empirical evidence, though the contributory factors identified within the three contexts may vary depending on the study (Awa & Ukoha, 2017). Nevertheless, TOE neglects the influence of social factors, such as friends, family, staff, and inter-organizational and individual factors, such as top management support and decision-maker-related attributes, which are essential in the SME context (Alkhalil et al., 2017; Parker & Castleman, 2009). Various studies have used TOE to explain IT/IS adoption in SMEs (Eze et al., 2019; Ramayah et al., 2016) as well as cloud computing adoption (Kim et al., 2017; Senyo et al., 2018).

### Diffusion of innovation (DOI)

The Diffusion of Innovation (DOI) framework, presented by Rogers (2010), serves as a theoretical foundation for IT/IS adoption studies at both individual and firm levels across various contexts (Salahshour Rad et al., 2018). DOI emphasizes two influential factors—innovation attributes and firm innovativeness. Innovation attributes such as relative advantage, compatibility, complexity, trialability, and observability are important in explaining the rate of individuals' adoption. Innovativeness is related to independent variables, such as leader characteristics, firms' internal structure, and external features (Hoonsopon & Puriwat, 2019, 2021). Similarly, the technology context of TOE is often linked to the five innovation attributes of DOI. The difference between the two is that TOE incorporates the external environment as having a positive or negative influence on technological adoption, whereas DOI does not consider external factors (Oliveira & Martins, 2010). Multiple studies have used DOI to explain IT/IS adoption in SMEs (Alam et al., 2011; Alshamaila et al., 2013; Oni & Papazafeiropoulou, 2014). Alam et al. (2011) investigate 200 SMEs in Malaysia to determine the significant factors that influence their e-commerce adoption. The authors identify five important aspects in this regard: relative advantage, compatibility, organizational readiness, owner‐manager's characteristics, and security. However, critics of Roger's theory argue that the DOI portrays diffusion as a linear process and fails to address important facets, such as institutional concepts (Beynon-Davies & Williams, 2003; Lyytinen & Damsgaard, 2001). Moreover, unlike those based on TOE, DOI-based analyses ignore environmental factors (Alkhalil et al., 2017).

### Institutional theory (INT)

Institutional theory (INT), proposed by Dimaggio and Powell (1983), argues that institutional environments are critical in shaping organizational structures and actions. INT identifies three types of pressures, namely, coercive—pressure from external stakeholders that a firm depends on; normative—pressure from social norms, values, and standards; and mimetic—pressures from demands of superior performance (Kung et al., 2015). According to Scott and Christensen (1995), institutional pressures erode existing organizational norms and practices. INT can help reinforce the TOE framework's theoretical strength and deep understanding of the environmental context and cloud software-as-a-service (SaaS) adoption (Oliveira et al., 2019). However, Yigitbasioglu (2015) found that INT has greater relevance in organizational transformation studies and is addressed less by cloud computing adoption scholars. Therefore, in line with Kung et al. (2015) and Martins et al. (2016), it is necessary to incorporate institutional factors when studying cloud-SaaS adoption in SMEs. Implementation of INT complements the use of TOE and DOI by providing a more holistic perspective of the effect of environmental pressures on the adoption of cloud accounting.

### Integrated theory and extended factors

According to Parker and Castleman (2009) and Alkhalil et al. (2017), one theory cannot explain all types of technology adoption. Thus, many researchers have integrated theories and/or extended factors to enhance our understanding of how a particular technology is accepted and used. A summary of previous literature on cloud computing adoption, accounting information systems (AIS), and digital technologies in SMEs is shown in Table 3. In addition, we present an overview of studies that use technological, organizational, environmental, and vendor- and owner-related factors to explain both SaaS and IT/IS adoption by SMEs in Table 4. This table was created from a literature review based on a Google Scholar search for the strings “SaaS” + “Technology Adoption” and “SMEs” + “Technology Adoption,” limiting the search to SJR Q1/Q2 journals published during 2015–2019.

**Table 3:** Previous studies on adoption of cloud computing, AIS, and IT in SMEs

Study	Scope of study				Theoretical model used			
	Domain	Organization	Country	Perspective	TOE and similar context	DOI	INT	Extended factors
*Cloud computing adoption (focusing on SaaS)*								
Kim et al. (2017)	Cloud-SaaS	SMEs	Korea	Owner‐Managers	√			√ (Risk and benefit)
Martins et al. (2016)	Cloud-SaaS	Firms in various industries	Portugal	IT	√	√	√	
Safari et al. (2015)	Cloud-SaaS	Firm in IT service industry	N/A	A group of experts	√	√		
Cho and Chan (2015)	Cloud-SaaS	Firms in various industries	Hongkong	IT	√ (Technology/ Organization)			√ (Vendor, Owner/Manager)
Kung et al. (2015)	Cloud-SaaS	Firms in manufacturing and retail industries	United States	IT		√	√	
Seethamraju (2015)	Cloud-SaaS (ERP)	SMEs	India	Owner‐managers and IT vendors	√ (Technology/organization			√ (Vendor)
Yigitbasioglu (2015)	Cloud in general	Firms in manufacturing and service industries	Australia	IT			√	√ (Owner/Manager)
Khayer et al. (2020)	Cloud in general	SMEs	Bangladesh	Owner‐managers	√			√ (Owner‐Manager)
Hiran and Henten (2020)	Cloud in general	Education institution	Ethiopia	Stakeholders	√	√		√ (Socio-Culture)
Alkhater et al. (2018)	Cloud in general	Firms in various industries	Saudi Arabia	A group of experts	√	√	√	√ (Trust, privacy, and physical location)
*Accounting information system (AIS) adoption*								
Dmour et al. (2016)	AIS	SMEs	Jordan	Owner‐managers	√			
Alamin et al. (2020)	AIS	N/A	Libya	Accountants			√	√ (e.g., Performance expectancy, Perceived technology fit)
*IT adoption in SMEs*								
Ramayah et al. (2016)	Website	SMEs	Malaysia	Owner‐managers	√	√		√ (Owner‐Manager)
R. Rahayu and Day (2015)	E-Commerce	SMEs	Indonesia	Owner‐managers	√			√ (Owner‐Manager)
Taiminen and Karjaluoto (2015)	Digital marketing channels	SMEs	Finland	Owner‐managers	√ (Organization/ Environment)			√ (Owner‐Manager)
Al-Bakri and Katsioloudes (2015)	E-Commerce	SMEs	Jordan	Owner‐managers	√ (Organization/ Environment			√ (Owner‐Manager)

**Table 4:** Previous studies that use technology, organization, environment, vendor, and owner factors

Factors	Description	Sub–factors
Technology (T)	Pool of internal and external technologies that are useful to the firm	Relative advantage (Martins et al., 2016; Safari et al., 2015; Yang et al., 2015) – Complexity (Kung et al., 2015; Martins et al., 2016; Safari et al., 2015) – Perceived benefit (Cho & Chan, 2015; Kurnia et al., 2015; Seethamraju, 2015) – Experienceability and trialability (Safari et al., 2015; Yang et al., 2015) – Compatibility (Safari et al., 2015; Yang et al., 2015) – Perceived risk (Cho & Chan, 2015) – Cost savings (Martins et al., 2016) – Simplicity (Yang et al., 2015) – Configurability (Seethamraju, 2015) – Observability (Safari et al., 2015) – Security and privacy (Safari et al., 2015)
Organization (O)	Key resources and nature of business	– Top management support (Cho & Chan, 2015; Martins et al., 2016; Van de Weerd et al., 2016; Yang et al., 2015) – Change management ability (Seethamraju, 2015) – Sharing and collaboration culture (Safari et al., 2015) – Organizational readiness (Van de Weerd et al., 2016) – Perceived organization resources and governance (Kurnia et al., 2015) – Technology competence (Martins et al., 2016) – IT readiness (Seethamraju, 2015) – IT infrastructure (Yang et al., 2015) – IT resource (Safari et al., 2015) – Gap in IT capabilities (Cho & Chan, 2015) – Business software fit (Seethamraju, 2015) – Sales volume of SMEs (Nair et al., 2019) – Business characteristics (Martínez-Román & Romero, 2017)
Environment (E)	Pressures and supports that exist in the business field	– Competitive pressure (Cho & Chan, 2015; Safari et al., 2015) – Normative pressure (Kung et al., 2015; Martins et al., 2016) – Mimetic pressure (Kung et al., 2015) – Coercive pressures (Martins et al., 2016) – Pressure from customers (Nair et al., 2019) – Competitor pressure (Yang et al., 2015) – Partner pressure (Yang et al., 2015) – Social influence (Safari et al., 2015) – Perceived environmental pressure (Kurnia et al., 2015)
Vendor (V)	Organization that offers Cloud–SaaS and IT/IS for SMEs	– Support and service quality (Cho & Chan, 2015; Seethamraju, 2015) – Perceived supporting services (Kurnia et al., 2015) – Reputation (Seethamraju, 2015) – Co-creation of value (Seethamraju, 2015)
Owner (O)	Individual who owns and operates an SME business	– Owners' IT ability and knowledge (Nair et al., 2019; R. Rahayu & Day, 2015; Taiminen & Karjaluoto, 2015) – Owners' innovativeness (R. Rahayu & Day, 2015; Ramayah et al., 2016) – Owners' IT attitude (Nair et al., 2019; Ramayah et al., 2016) – Owners' attitude toward change (Alharbi et al., 2016) – Attitude of management toward ownership and control (Cho & Chan, 2015) – Owners' perception (Al-Bakri & Katsioloudes, 2015) – Owners' IT experience (R. Rahayu & Day, 2015) – Owners' age (Nair et al., 2019) – Owners' personal characteristics (Martínez-Román & Romero, 2017)

The data in Tables 3 and 4 indicate that many studies have attempted to integrate the strengths of traditional adoption theories to provide clearer decision-making lenses, for instance, TOE–DOI–INT (Alkhater et al., 2018; Martins et al., 2016), traditional adoption theories with vendor factors (Cho & Chan, 2015; Seethamraju, 2015), and traditional adoption theories with owner‐manager factors (Khayer et al., 2020; Ramayah et al., 2016; Yigitbasioglu, 2015). In the case of SME studies, the owner–manager is a crucial factor, contributing to the success or failure of adoption. Furthermore, the vendor factor is particularly relevant for cloud accounting, which employs a vendor-owned model.

## Methodology and data collection

This study protocol has been approved by the Research Ethics Review Committee for Research Involving Human Subjects on August 7, 2020.

### Research methodology

In this study, we followed an exploratory, qualitative, multiple-case study approach to investigate how technology‐organization‐environment‐vendor‐owner (TOEVO) factors influence cloud accounting adoption in Thai SMEs. We prefer a qualitative over a quantitative method for three main reasons. First, cloud accounting is relatively new in the field of business practices and accounting (Rindasu, 2017). Therefore, a qualitative approach based on semi-structured interviews is more appropriate for exploring and understanding various aspects related to the concept (Das & Dayal, 2016). Second, a qualitative approach, through the use of interactive conversation and open-ended questions, enables researchers to better understand different informants' behaviors, experiences, and reasons for adopting cloud accounting (Rahman, 2017). Third, the multiple-case study method allows for in-depth investigation of the current state of cloud accounting in real-world settings (Yin, 2014).

With the case study method, we intended to determine why the adoption rate of cloud accounting in Thailand is relatively low, and whether and how TOEVO variables influence the adoption decision, especially following the COVID-19 pandemic. We conducted semi-structured interviews with SME owners, cloud accounting vendors, and a group of experts (including professional accountants) to gain a business perspective, which is vital but less explored by researchers (Senyo et al., 2018).

### Case study organization and informants

This study uses a triangulation of sources, that is, an examination of the same information from different data sources (experts, cloud accounting vendors, and SMEs owners) to increase the richness and validity of its findings. Data were gathered from 17 informants who were involved in various SMEs' decision-making stages, such as research, selection, and adoption of cloud accounting. The informants include six experts, five cloud accounting vendors, and six SME owners. According to Felix and Hunter (2002), a sample size of 15–25 informants is sufficient for qualitative research that is similar to our study. A detailed summary of informants' profiles is provided in Table 5.

**Table 5:** Informant profile

Group	Informant code	Role	Experience	Sex	Age	Education
Expert	EX1	– Managing director	– 15 year experience as accountant – Using cloud accounting for SME clients and for internal use	F	40–50	Master’s degree
Expert	EX2	– Top management in government agency	– 20 year experience in IT and start-up business	M	40–50	Master's degree
Expert	EX3	– Academic director of entrepreneurship development institute – President of technology-related association – SaaS CEO/Founder	– 15 year experience in start-ups, cloud accounting, digital innovation, and platform strategy	M	40–50	Doctoral degree (PhD)
Expert	EX4.1	– Senior technical expert	– 30 year experience in technology for SMEs –Government officer	M	50–60	N/A
Expert	EX4.2	– Industrial analyst	– 5 year experience in e-commerce for SMEs – Government officer	F	30–40	Master's degree
Expert	EX5	– Manager of commercial bank that operates SaaS recommender system for SMEs	– 5 year experience in SME banking and insurance	M	30–40	Master's degree
Vendor	VE1	– CEO/founder of cloud accounting company	– Background in IT and small business	M	30–40	Master's degree
Vendor	VE2	– CEO/founder of cloud accounting company	– Background in accounting	M	30–40	Master's degree
Vendor	VE3	– CEO/founder of on-premise accounting software and accounting service company; cloud accounting re-seller	– Background in accounting	F	50–60	Master's degree
Vendor	VE4	– CEO/founder of cloud accounting and accounting services company	– Background in accounting	M	40–50	Master's degree
Vendor	VE5	– CEO/founder of on-premise accounting software and cloud accounting company	– Background in IT	M	50–60	Master's degree
Owner	OW1	– CEO/founder of cosmetic company – Company aged 0–3 years – Family business – B2B & B2C	– A few years' experience in banking – Experience managing others' family business (hotel)	F	30–40	Master's degree
Owner	OW2	– CEO/founder of food manufacturing company – Company aged 4–6 years – Family business – B2B & B2C	– A few years' experience in food & beverage company	M	30–40	Master's degree
Owner	OW3	– COO/founder of food delivery service – Company aged 4–6 years – Startup business – B2C	– A few years' experience in international food & beverage company	F	30–40	Master's degree
Owner	OW4	– CFO/founder of marketing agency – Company aged 7–9 years – Business with friends – B2B	– Experience managing others' business (food & beverage) – Background in engineering	M	30–40	Bachelor's degree
Owner	OW5.1	– CEO of engine trading company – Company aged 7–9 years – Family business – B2B & B2C	– Experience managing others' family business (Air conditioning distributors)	M	30–40	Bachelor's degree
Owner	OW5.2	– CFO of engine trading company – Company aged 7–9 years – Family business – B2B & B2C	– Experience managing others' family business (Air conditioning distributors) – Background in finance	F	30–40	Master's degree

The semi-structured interviews were conducted in Thailand from August to September 2020, 3 months after the first nationwide lockdown in response to COVID-19 in Thailand. We conducted in-depth interviews with the informants, each of which lasted 45–75 min. The interviews where held either via Zoom video conference or face-to-face while wearing a face mask, depending on the informant's preference. During the data collection process, we began by asking “What is cloud accounting/cloud computing?” to ensure that all informants were familiar with the concepts of cloud accounting/cloud computing. Based on the literature review, the informants were broadly asked questions about (a) their background/institutions and views on cloud accounting in Thailand; (b) SMEs' reasons and processes for deciding to select/not select cloud accounting; and (c) factors that led to cloud accounting adoption. With the permission of the informants, the interviews were tape-recorded, fully transcribed, and analyzed based on the qualitative content analysis approach. Content analysis was chosen due to its suitability for exploratory research (Lune & Berg, 2017).

## Findings and analysis

In this section, we present the significant findings obtained from our semi-structured qualitative interviews.

### Why do SMEs adopt cloud accounting?

Cloud accounting in Thailand is in the early stages of adoption, as confirmed by 10 out of 11 expert and vendor informants. The existing adoption rate is estimated at 50,000–70,000 companies, or 5–10% of juristic-entity SMEs, as per the following interview extracts:

Thailand has a relatively low user adoption rate. Most SMEs set their own manual accounting system or outsource accounting services. (EX4.2)

SaaS is perceived as very new by Thai SMEs. Current adoption should be around 10% of juristic SMEs or 50,000 firms, so the untapped potential is very large, approximately 90%. (VE2)

Respondents in our study reported that SMEs' decision to adopt cloud accounting was triggered by perceived internal needs and problems rather than external information about cloud accounting (*n* = 14). However, the reasons for adoption varied; the most frequently cited were as follows: to improve efficiency (*n* = 7), to obtain real-time data (*n* = 4), and to increase mobility (*n* = 3), as illustrated by the following excerpts:

We aim to reduce the number of workers, manual work, and paperwork in all processes. (OW1)

The main reason is real-time financial data, which is normally missing. With traditional accounting software, sometimes, SMEs do not even know who owe them money. With cloud accounting, they can better track accounts payable, accounts receivable, expenses, revenue, and margins in detail. (VE4)

Mobility is the reason why SMEs decide to adopt cloud accounting. After sales meetings, SMEs can immediately issue a purchase order from their mobile or tablets and send it to the customer via the LINE application. They have no need to return to their offices. (EX5)

### How do SMEs start adopting cloud accounting?

To facilitate the successful adoption of cloud accounting, the involvement of the SME's owner would be critical. In Thailand, SME owners are decision makers who are proactively involved in the end-to-end adoption process—initiation, evaluation, trial, selection, and acquisition—although this practice is quite unusual in other countries. Owners' decision-making is a nonlinear, ongoing process with several feedback loops. Before adopting new technology, they also actively seek strategic advice from external influencers, such as peers, friends, family, professional accountants, government units, and IT consultants, as well as internal influencers, such as internal accountants and co-owners.

A long time ago, I had tried a cloud accounting service, but the features in those days were poor. Therefore, I abandoned the adoption plan. A couple of years later, I heard from my peers who are currently using it that the features had improved. They recommended it to me. Then, I started searching for the available cloud accounting services again and applied for a trial to see whether it fit with my company's needs. (OW4)

One of my friends recommended cloud accounting, and I signed up for the free trial and found it easy to use. I later persuaded my husband to adopt it for our company. (OW5.2)

When SMEs make adoption decisions, they assess the perceived advantages of cloud accounting and the adoption costs by comparing cloud accounting with the current arrangement or available tools, such as Microsoft Excel and on-premise accounting software.

Adopters can be categorized into two groups. First, Microsoft Excel users who decide to move to proper online accounting software. Second, on-premise accounting software users whose scope of accounting has become more complex and are looking for a new solution that better serves their needs. Both groups do some research to compare the pros and cons of the available options fit for them. (VE2)

If SMEs preferred mobility, they would choose the cloud, but if they are uncomfortable with the monthly fee model and worry about security, they would choose on-premise software. (EX3)

### What are the driving factors?

We evaluate the driving factors most frequently mentioned by informants (at least nine informants). Each factor has a varying influence on cloud accounting adoption, as follows:

#### Technological context

*(* +*) Relative advantage.* In our case study, relative advantage has a positive impact on cloud accounting adoption. Most informants indicated that relative advantage is a crucial factor in every IT initiative that SMEs decide to pursue, including cloud accounting. Some of these relative advantages include efficiency, accessibility, speed, accuracy, and professionalism, which ultimately lead to cost savings and improved profitability for SMEs. Below is a selection of the informants' responses:

Previously, SMEs lost important documents and wasted time searching for missing documents for the accounting service company. If SMEs store all documents on the cloud, the process of preparing financial reports and tax submissions by the accounting service company would be quicker, easier, and less time-consuming. (EX5)

I can use my smartphone to send sales quotations prepared in a professional format from wherever I am. Customers often shop around, so sending a sales quotation quickly will help us get ahead of our competitors. (OW5.1)

Sometimes, business owners are not aware of their current business performance and do not have a holistic view of individual customers. With cloud accounting, SME owners will have a better understanding of their current accounts receivable, accounts payable, and profitability in real time. (VE4)

Compared to other accounting methods, the benefits of cloud accounting include the ability to (1) improve collaboration and information visibility between SMEs and the accounting service company, (2) issue purchasing orders and invoices to customers, record business expenses, and receive payments anywhere and at any time, and (3) access up-to-date information on financial performance and the customer.

*(-) Complexity.* Although the adoption of digital technologies by SMEs is considered complex in many countries, the situation in Thailand is much more difficult, as many of our informants noted. This is because Thai SMEs are in the early stages of IT-enabled business transformation. The informants stated that the vast majority of Thai SMEs preferred traditional methods of recording accounting transactions, prepared by their outsourced or internal accountants. Early adopter SMEs typically relied on external consultants, such as accounting service companies, peers, and vendors when adopting cloud accounting. They perceived cloud accounting as involving a “user-friendly interface,” “easy deployment and maintenance,” and “less work of preparing budgets, financial statements, and cash flow statements,” as illustrated by the following excerpts:

A simple usable UX/UI is one of my top selection criteria. (OW2)

Cloud accounting makes accounting easy for the company without a dedicated accountant or accounting experience. (OW3)

In particular, informants noted that the perception of complexity was inversely related to SMEs' extent of experiences with other cloud-based solutions and digital technologies in general.

*(* +*) Compatibility.* Herein, compatibility refers to the degree to which cloud accounting, as an innovation, is seen as consistent with the existing values, needs, and past experiences of SMEs. Several informants confirmed the importance of introducing digital-enabled business practices that are compatible with current organizational practices. There are different needs within the SME segment; therefore, cloud accounting has been adapted in different ways based on the size and scope of operations, as illustrated in the following excerpt:

Cloud accounting is typically for SMEs with a non-complex business model, such as a trading company, general service company, or small agency. It is not suitable for companies with many subsidiaries that need to comply with advanced accounting rules. Therefore, in my case, I use cloud accounting for quotations and invoice processing only. My accountancy team handles accounting separately to ensure accuracy in financial statements. (OW4)

Based on the interviews, the following three conditions within firms are identified as particularly well-suited to embracing cloud accounting: (1) having a simple business structure and operations, (2) being equipped with computer and Internet access, (3) and engaging with accounting service companies that have adopted automation and cloud accounting software.

#### Organizational context

*(* ±*) Firm characteristics*. In this study, the term “firm characteristics” refers to firm size and nature of business. Firm size is defined by the number of employees and annual revenue, whereas nature of business refers to industry type, the business model, geographical scope, and taxation requirements. Below is a selection of the informants' responses on the firm characteristics that influence adoption:

In case of mom-and-pop stores, they do not benefit from cloud accounting. However, if they want to grow the business or set a company standard, cloud accounting is the best fit for their businesses. (EX2)

Most adopters are juristic-entity SMEs, because they are required by Thai law to file and pay monthly taxes. If they are late or fail to meet a required legal obligation, penalties will apply immediately. (VE1)

Most adopters are business-to-business (B2B) SMEs that operate nationwide. SME owners use cloud accounting for tracking salesperson records and available stock with greater ease. (EX1)

In general, the informants concluded that cloud accounting is relatively important for the trading and service-type industries. Registered SMEs, B2B SMEs, and SMEs with a wider geographic presence were more willing to adopt cloud accounting.

*(* ±*) Technology competence*. Technology competence refers to three aspects: (1) digital technology knowledge—the degree to which SMEs realize the ability of existing and emerging digital technology, (2) digital technology utilization—the extent to which SMEs employ digital technology to enhance their effectiveness and decision-making, and (3) digital technology facility—a set of tools that contribute to SMEs' digital technology utilization. We find that prior experiences and success in digital technology adoption are a source of digital technology knowledge and increase its utilization. At present, cloud accounting relies mainly on having an Internet connection and Internet-enabled devices. Poor and slow Internet connections can affect the work methods of rural SMEs.

SMEs that rarely use technology are reluctant to adopt cloud accounting. (VE3)

They selected SaaS, because they have limited technology competence. (VE4)

This study portrays two different views on technology competence. On the one hand, it is believed that cloud accounting fits SMEs particularly well as they usually have limited technology competence and resources (*n* = 9). On the other hand, some informants argue that if the level of technology competence in SMEs is too low—if they have no computer or Internet access—cloud accounting adoption is simply not possible (*n* = 8).

#### Environment

*(* +*) Coercive pressure*. At present, Thai government agencies, such as the Digital Economy Promotion Agency, Department of Industrial Promotion, and Department of Business Development are consistently promoting and enforcing digital technology adoption among SMEs. This is done through government policies, such as a 200% tax deduction scheme up to 100,000 THB for the purchase of digital technology (including cloud accounting), a grant of up to 10,000 THB to support SMEs' software purchasing needs—including fees for cloud accounting, software training, and consultation—and a free e-accounting program for SMEs and electronic tax return filing platforms, as confirmed by the following quotes:

Right now, we use webinars to connect with SMEs. Before the COVID-19 pandemic, we arranged face-to-face consultations with SMEs seeking software-related advice and government grant information. This year, we have 3,500 grants available, valued up to 10,000 THB each. (EX2)

Government policy increasingly favors SaaS adoption in SMEs; for instance, the DBD e-filing system—submitting financial statements of a company established under Thai law online. (VE3)

According to expert groups, coercive forces are mainly triggered by regulations associated with tax-related benefits and government sponsorship. However, vendor and owner groups perceived current government support as being less attractive and rather proposed promotion of long-term cloud accounting usage, such as speedy and streamlined loan scheme applications for SMEs that periodically synchronize their financial information with the cloud accounting platform.

*(* +*) Normative pressure*. In this research, sources of normative pressure include industry norms and customer expectations. These norms were created through training, social networks, and professional processes. Some informants mentioned that normative pressure could also be traced to other bodies that have successfully employed cloud accounting.

People in the digital startup industry have a sense of getting it done fast and online only. That's why I chose cloud accounting since Day 1. (OW3)

First, the owners attended SME trainings and seminars. Then, they realized the importance of digital tools and finally asked us for an adoption consultation. (EX4.1)

*(* +*) Mimetic pressure*. Some SMEs felt pressure to adopt cloud accounting, typically because of its adoption by their competitors or other similar organizations. Our informants determined that mega trends and competitive intensity also encourage SMEs to adopt cloud accounting. As increasing numbers of SMEs adopt cloud accounting during the COVID-19 pandemic, mimetic pressure to change will increase rapidly for those who do not.

SMEs were forced to adopt cloud accounting due to mega trends, such as globalization and competitive rivalry among the firms in an industry. (VE4).

After reviewing the vendor's website, I felt its targets SMEs similar to mine. If others can use it, I should use it, too. (OW2)

This finding further indicates that pressure from competitors is more impactful than that from other stakeholders.

*(* +*) COVID-19 pressure*. All informants perceived that the COVID-19 pandemic has accelerated the digital transformation of SMEs. Cloud accounting awareness and adoption has been consistently increasing post-COVID-19. Interviewee comments often highlighted that SMEs with cloud accounting have been able to adapt readily to the lockdown, while others are unable to operate effectively. As mentioned by informants, cloud accounting can help in avoiding physical contact, accessing up-to-date financial information remotely, and integrating with other systems, such as e-commerce platforms, payroll, and warehouse management.

The adoption is rising. SMEs want to use cloud accounting as soon as possible, because they saw that their competitors who already adopted cloud accounting could run their business more smoothly during the COVID-19 lockdown. (EX2)

COVID-19 and work-from-home policies are raising the awareness of SMEs who are using on-premise software. (VE4)

The informants concluded that the ongoing COVID-19 pandemic has pushed SMEs to opt for remote working, and cloud accounting has contributed to maintaining business continuity in this period.

#### Vendor

*(* +*) Vendor support and service quality*. Cloud accounting vendors provide more than software and hardware; they also deliver expertise in a specific accounting area that SMEs feel can augment their accounting knowledge, process, and system. Vendor support and service quality refer to vendor promotion, training workshops, and technical support. In Thailand, online and offline training is regularly provided by local vendors:

I think SMEs decide based on vendor support and functionality. Old SMEs in particular prefer a vendor that can answer their questions and resolve their issues quickly. (EX2)

I first chose this vendor because of special promotion—50% discount, equal to just 3,000 THB per year. It is not expensive for an initial-stage business-like mine. (OW1)

Vendor support and service quality are key considerations, since adopting cloud accounting requires strong commitment between the vendor and SMEs. (VE5)

The interview outcomes indicate that cloud accounting vendors play an important role in helping SMEs to implement cloud accounting and facilitating their use of this technology while aiding resolution of both technical and accounting issues.

#### Owner

*(* +*) Owner's support*. In our study, the decision to adopt cloud accounting made entirely by SME owners, who usually make all business decisions, from daily operations to future investments. To facilitate successful implementation, SME owners are proactively involved in the adoption process, including the initiation, evaluation, trial, selection, and acquisition of software. Proactive owners are convinced of the benefits of cloud accounting, and likely to support it.

Owner's participation and strong desire to use are key contributors to the digital technology adoption success within SMEs. (EX4.2)

The owner is the key decision maker to adopt or not adopt cloud accounting. (OW1)

All six SME owners who were involved in the entire adoption process perceived cloud accounting adoption and usage positively.

*(* +*) Owner's attitude.* Attitude toward change is related to owners' change competency, such as taking risks, emphasizing goal achievement, and being socially active. SME owners that have a greater tolerance for risk and change are more likely to accept cloud accounting. For instance, OW3 was a visionary and passionate about the future direction of her business. She was a risk taker, judging by her decision to adopt Xero, a New Zealand-based cloud accounting provider not commonly used in Thailand.

Six years ago, we decided to use cloud accounting though there was only Xero available. We are possibly the first Thai user of Xero. (OW3)

The owner's positive attitude toward change is critical. If the owner prefers not to change, new technology adoption is not possible. (OW4)

Ten out of seventeen informants believed that the SME owner's attitude was critical for any IT/IS adoption success, including cloud accounting.

*(* +*) Owner's innovativeness.* Owner's innovativeness appears to be another determinant of cloud accounting adoption. Innovative owners prefer to apply distinctive and risky solutions, such as cloud accounting that modify the traditional structure. As all owner informants mentioned, they always seek, support, and contribute to innovative activities, which results in enhancing the firm's efficiency and competitiveness. New technology and research and development (R&D) are common in these firms.

To support operation, we develop our own task-tracking software on the cloud for internal and customer use. We never use the local server in any areas. (OW4)

Owners' innovativeness is very important. If they are tech-savvy, they normally choose online, rather than offline solutions. (VE2)

These results suggest that the innovativeness of the owner influences the cloud accounting adoption decision in SMEs.

## Discussion

In this section, we first discuss why and how SMEs adopt cloud accounting in Thailand and then elaborate on the influence of the TOEVO factors on cloud accounting adoption.

### Why SMEs adopt cloud accounting

Before examining why SMEs adopt cloud accounting, it is worthwhile understanding the state of cloud accounting in developing countries, particularly in Thailand. Our study indicates that most Thai SMEs did not use cloud accounting before the COVID-19 pandemic. They were reluctant to adopt cloud accounting due to their reliance on traditional processes centered around paperwork and desktop-based software. This is in line with Nyathi et al. (2018), who found that 81% of SMEs in Zimbabwe use manual record-keeping systems and Harash (2017), finding that most Iraqi SMEs have not adopted proper accounting information systems.

Our cross-case analysis indicates that the intention to adopt cloud accounting was initially triggered by internal needs to improve business activities, rather than by external factors. The key rationales are to improve efficiency through lower operating costs and reduced error, stay competitive in a data-driven world, and increase mobility in response to the pandemic. As a top priority, SMEs' higher efficiency is derived from lower manual workload, no upfront IT investment, fewer workers, and minimized paperwork, as well as online collaboration between co-owners, SMEs and customers, and SMEs and accounting service companies. This is a key rationale in developing countries and has also been reported in other IT/IS adoption studies (Thu Ha et al., 2020).

### How SMEs adopt cloud accounting

We find that, unlike large established organizations, SMEs take an informal adoption approach to cloud accounting. This finding is aligned to that of Carcary et al. (2014), which states that SMEs do not emphasize creating or utilizing proper cloud readiness assessment criteria. Moreover, as SMEs are smaller and family- or friends-owned businesses, the owners normally make all decisions based on their individual traits, including attitude, knowledge, and experience. Key owner traits are owners' innovativeness and their attitude toward change. This observation confirms the findings of previous cloud and SME technology adoption studies (Alharbi et al., 2016; Cho & Chan, 2015; Rahayu & Day, 2015; Ramayah et al., 2016; Seethamraju, 2015).

During the adoption process, SME owners use the Internet to investigate which tool would suit their situation, review its accounting functionality (including user experience and user interface), and then compare costs with other available tools. For micro and small businesses, low price and ease of use are more important than complete functionality. These results are in line with the findings of Levenburg (2005). Although price is an SME priority, financial constraints for cloud accounting and other IT-related resources is of lesser concern. Our finding contradicts that of other SME IT/IS adoption studies that financial constraints are one of main IT/IS implementation challenges in developing countries (Apulu et al., 2011; Hernandez, 2020; Kabanda et al., 2018). This is because cloud accounting platforms offer affordable pay-per-use services with flexible payment options, allowing SMEs to pay only for the functions they need, making it a cost-effective solution (Kim et al., 2017).

This study shows that SME owners are not only decision makers but also initiators, trial users, evaluators, and executors during the adoption process, confirming that owner commitment is essential in the implementation of accounting-related information systems (Rahayu, 2012). Furthermore, we found that owners' adoption decisions were also influenced by various stakeholders, such as peers, friends, family, professional accountants, government units, vendors, and IT consultants, both online and offline. Similarly, Nguyen et al. (2015) show that the assistance of external IT experts during the adoption process helps to reduce failure risk within SMEs.

### Conceptual model based on TOEVO factors

The study's findings reveal that vendor, owner, technological, organizational, and environmental factors have a positive effect on cloud accounting adoption in SMEs.

**Fig. 1:**
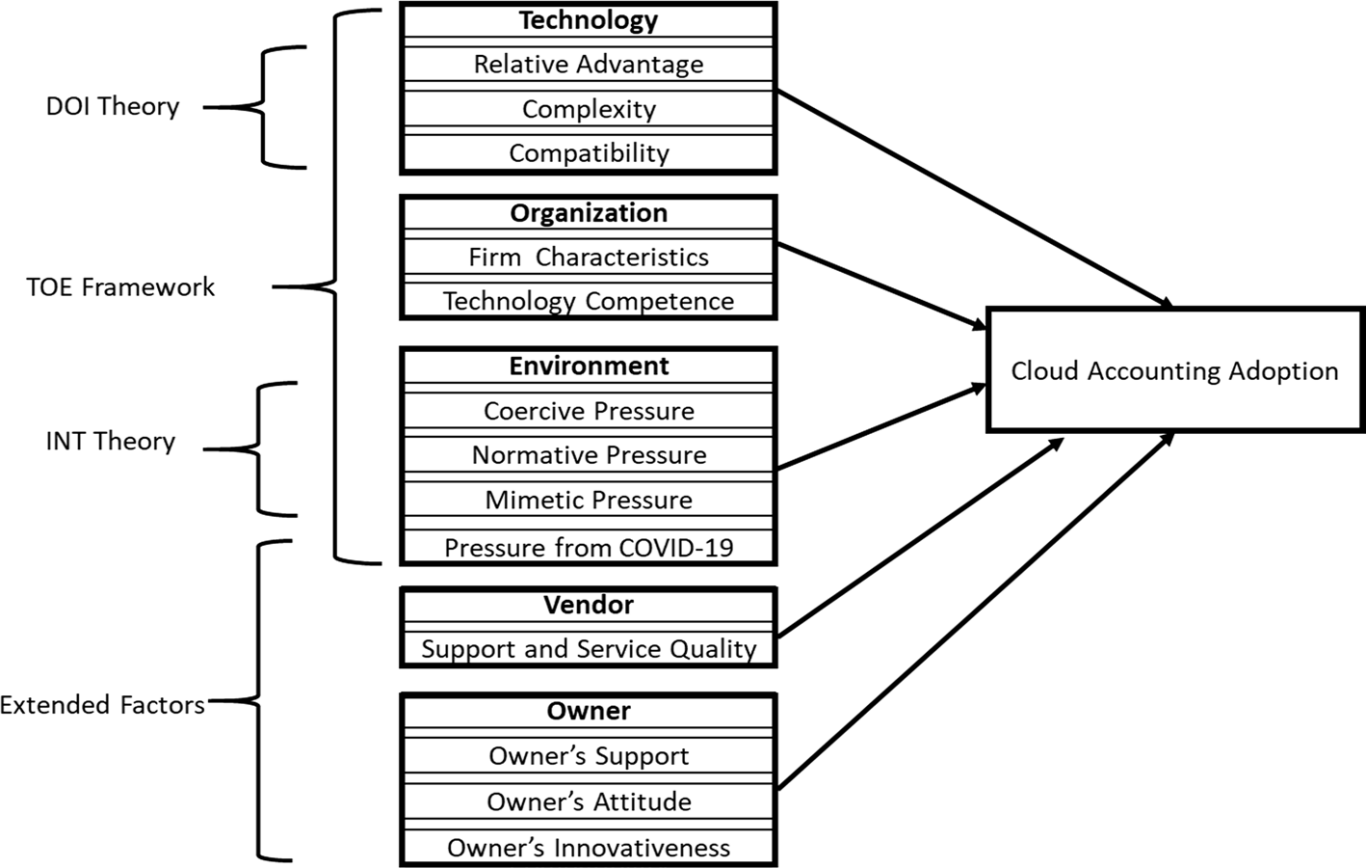
HYPERLINK "sps:id::fig1||locator::gr1||MediaObject::0" Technology–organization–environment–vendor–owner–cloud accounting adoption framework for small and medium enterprises

An integrated conceptual model through the lens of extended TOE–DOI–INT, depicting the relationship between relevant factors and SMEs' decision to adopt cloud accounting, is presented in Fig. 1.

With regard to the environment, all determinants confirmed the findings of previous IT/IS adoption studies (Kung et al., 2015; Martins et al., 2016), except for pressure from the COVID-19 pandemic, which is an additional determinant identified by this study. Our findings indicate that work-from-home arrangements and the national lockdown have accelerated the demand for cloud-based solutions from SMEs, especially cloud accounting. This is in line with outcomes of a recent survey, which confirmed that the COVID-19 pandemic has become a more important determinant in SMEs' cloud-specific purchasing decisions since 2020 (International Trade Center, 2020).

Vendor support and service quality are important factors from our study that align with a previous study conducted by Lewandowski et al. (2013), who noted that vendor support during and after implementation proved valuable to SMEs. Furthermore, cloud accounting vendors often understand SMEs' unique needs and, therefore, target their marketing toward adoption by SMEs. This finding contradicts Stockdale and Standing (2004), who indicate that SMEs face obstacles as IT vendors often restrict their marketing to larger firms.

## Conclusions and implications

In terms of theoretical implications, this is presumably the first study to explore cloud accounting adoption after the COVID-19 pandemic. By integrating TOE, DOI, INT, and extended factors, this study has shown that five aspects—technological, organizational, environmental, vendor-, and owner-related—are interconnected and affect SMEs' adoption and use of cloud accounting in the COVID-19 context. This paper triangulates sources of information, including experts, vendors, and owners, to better understand the organizational context of SMEs. Thus, it contributes to theory regarding cloud computing adoption and SMEs.

Furthermore, this paper provides significant practical implications for the research community, policymakers, cloud accounting vendors, and SME owners, in terms of formulating better approaches for cloud accounting adoption after the COVID-19 pandemic. The results suggest that policymakers can promote cloud accounting adoption along with faster loan scheme applications, rather than tax benefit policies (see Coercive pressure). Moreover, vendors should focus on SMEs' particular characteristics and needs. By contrast, SMEs should determine the organizational fit of the cloud accounting platform and utilize cloud accounting to its full potential, for instance, in cashflow management and integration with other cloud-based open platforms for inventory management, e-commerce platforms, and point-of-sale software.

As cloud accounting is relatively new in Thailand, this study focuses on its adoption and initial usage by SMEs. For future research, these findings are potentially transferable to other types of organizations and beyond the geographic restrictions explored initially. That is, to address this study's limitations, the TOEVO framework developed here requires further investigation and testing using larger sample sizes, a broader range of SME characteristics (e.g., different organizational size and nature of business), and more locations. Future studies might also examine the diffusion of cloud accounting across time, given that factors influencing initial adoption could possibly change during the routinization and infusion stages.

## Data Availability

All data generated or analyzed during this study are included in this published article and its supplementary information files.
